# Painful and recurring injection site reaction to alirocumab and evolocumab in a young woman with familial hypercholesterolemia and effective therapeutic alternative based on inclisiran: a case report

**DOI:** 10.3389/fcvm.2023.1181720

**Published:** 2023-06-23

**Authors:** Massimiliano Allevi, Silvia Sarnari, Federico Giulietti, Francesco Spannella, Chiara Di Pentima, Riccardo Sarzani

**Affiliations:** ^1^ Internal Medicine and Geriatrics, IRCCS INRCA, Ancona, Italy; ^2^ Department of Clinical and Molecular Sciences, University “Politecnica delle Marche”, Ancona, Italy

**Keywords:** familial hypercholesterolemia, polygenic hypercholesterolemia, injection site reaction, PCSK9-Inhibitor, inclisiran

## Abstract

A 28-year-old woman with autosomal dominant familial hypercholesterolemia (FH) with a probable coexistent polygenic contribution causing very high low-density lipoprotein-cholesterol (LDL-C) levels, started therapy with the proprotein convertase subtilisin/kexin type 9-inhibitor (PCSK9i) alirocumab, in addition to high-intensity statin plus ezetimibe. Forty-eight hours after the second injection of alirocumab, the patient developed a painful palpable injection site reaction (ISR) that recurred after the third administration of the drug. Treatment was then switched to evolocumab, another PCSK9i, but the patient had an ISR with similar features. The most conceivable cause of the ISR was a cell-mediated hypersensitivity reaction to polysorbate, an excipient contained in both drugs. Although ISR after PCSK9i administration is usually transient and does not compromise the continuation of treatment, in this case the recurrence of such side effect in an exacerbated way led to treatment withdrawal, with a subsequent re-exposure to increased cardiovascular (CV) risk. As soon as it became available in clinical practice, the patient started treatment with inclisiran, a small interfering RNA targeting hepatic PCSK9 synthesis. No adverse events were reported after inclisiran administration and LDL-C levels decreased significantly, confirming the evidence that this innovative approach to hypercholesterolemia is a safe and effective resource in patients at high CV risk who cannot achieve LDL-C goal with conventional lipid-lowering therapies and antibody-based PCSK9i.

## Introduction

Familial hypercholesterolemia (FH) is a common monogenic autosomal codominant dyslipidemia with a prevalence of 1 in every 200–250 individuals, characterized by increased low-density lipoprotein-cholesterol (LDL-C) levels and high risk of atherosclerotic cardiovascular disease (ASCVD) (1). Most of causative mutations reported in FH are loss-of-function mutations in the LDL receptor (LDLR) gene. Timely diagnosis is required and lipid-lowering treatment should be initiated as soon as possible to reduce the risk of premature ASCVD (2). The Dutch Clinic Lipid Network (DCLN) score is a validated and widely used instrument for the clinical diagnosis of FH. In Italy, the “Società Italiana per lo Studio dell’Aterosclerosi” (SISA) foundation set up a nationwide network named “Lipigen” (LIpid transPort disorders Italian GEnetic Network) to allow the genetic diagnosis in patients with a definite or probable FH according to the DLCN criteria (DLCN ≥6 points) (3). A pathogenic mutation can be detected in 40%–80% of clinically diagnosed patients with FH. In the other cases, elevated LDL-C levels might have a polygenic cause. Polygenic hypercholesterolemia (PHC) is determined by several common LDL-C-raising single nucleotide polymorphisms (SNPs) affecting multiple loci (4). FH and PHC are not mutually exclusive, but they can coexist in the same patient. Indeed, even in patients with a genetically diagnosed monogenic FH, a substantial polygenic contribution may subsist, contributing to the phenotypic variability observed in patients with the same FH-causative mutation. According to the most recent European guidelines for the management of dyslipidemias (5), treatment with a proprotein convertase subtilisin/kexin type 9-inhibitor (PCSK9i) is recommended in FH patients if the LDL-C goal is not achieved after the administration of a high-intensity statin plus ezetimibe. Currently, two fully human monoclonal antibodies (mAbs) are available in clinical practice to inhibit PCSK9: alirocumab and evolocumab, approved for self-administration biweekly by the subcutaneous (SC) route. Very recently, inclisiran has been introduced in clinical practice, offering a new strategy for PCSK9 inhibition through a different mechanism of action based on RNA interference (6). Indeed, inclisiran is a small interfering RNA (siRNA) targeting hepatic PCSK9 synthesis, administered twice-yearly by the SC route. Alirocumab, evolocumab and inclisiran, in addition to a significant reduction of LDL-C levels, confer a further cardiovascular (CV) benefit by lowering lipoprotein(a) [Lp(a)], a lipoprotein linked to increased risk of ischemic CV disease (7).

In this case report, we describe the impossibility to treat a young patient, suffering from heterozygous FH (HeFH) characterized by very high LDL-C and Lp(a) levels, with PCSK9i mAbs, due to the development of painful and recurrent injection site reaction (ISR) to both drugs. This is a very rare case in medical literature, since ISR after PCSK9i mAbs administration is usually transient and does not compromise the continuation of treatment (8). On the contrary, the recurrence of such side effect in an exacerbated way led to treatment withdrawal in our case, with a subsequent re-exposure to an increased risk of future ASCVD. Nevertheless, the very recent introduction of inclisiran for clinical use allowed us to safely and effectively treat this patient, leading to a significant reduction of both LDL-C and Lp(a) levels.

## Case description

### Patient information and clinical findings

In February 2021, a 28-year-old woman was referred to our Hypertension Excellence Centre of the European Society of Hypertension (ESH) and National Reference Centre of the “Lipigen” Network for Dyslipidemia in Ancona, Italy, for the management of a severe hypercholesterolemia. In the absence of any lipid-lowering treatment, the patient had the following lipid values: total cholesterol (TC) 445 mg/dl, high-density lipoprotein-cholesterol (HDL-C) 54 mg/dl, triglycerides (TG) 124 mg/dl and LDL-C 364 mg/dl [calculated using the modified Friedewald formula according to Martin et al. (9)]. Moreover, the patient had high Lp(a) levels (178 mg/dl), an additional known risk factor for ASCVD. Her family history included two older brothers with known premature coronary heart disease (CHD) and a first degree relative (mother) with known LDL-C >95th percentile by age and sex (240 mg/dl without any lipid-lowering treatment). On physical examination, the patient had bilateral Achilles tendon xanthomas, a typical finding in severe hypercholesterolemia (10). She was a moderate cigarette smoker (about 10 cigarettes per day). Secondary causes of hypercholesterolemia, such as diabetes mellitus, dysthyroidism, renal or hepatic dysfunctions, were excluded. Moreover, she was not taking systemic corticosteroids or estrogens. She did not suffer from hypertension, and she did not have history of ASCVD; carotid ultrasound did not detect atherosclerotic plaques, while coronary arteries calcium score was 0.

### Genetic analysis

The DLCN score was 15, a value indicating a definite clinical diagnosis of HeFH. Therefore, a genetic testing was performed through the “Lipigen” Network. The genetic analysis tested positive for a variant c.1374_1375del, p. (Arg458SerfsTer8) in the LDLR gene in heterozygosis, classified as pathogenic for FH and resulting in a null allele. Furthermore, we applied the equation described by Talmud et al. (11, 12), that includes 12 SNPs based on Global Lipid Genetic Consortium data (13) to detect the probability of PHC. According to this equation, values equal to or higher than 1.09 indicate a high probability of PHC, while values equal to or lower than 0.73 indicate a low probability of PHC. The score of our patient was 0.94, a value consistent with a substantial contribution of a polygenic cause. By contrast, the score of the patient’s mother, that tested positive for the same pathogenic mutation in the LDLR gene, was 0.7.

## Diagnostic assessment

### Therapeutic intervention

The primary goal of lipid-lowering therapy is to reach LDL-C levels below a certain threshold based on the individual CV risk. According to the most recent European guidelines for the management of dyslipidemias (5), patients suffering from HeFH are at least at high CV risk, which means that LDL-C should be lowered below 70 mg/dl. Therefore, our patient, whose baseline LDL-C was 364 mg/dl, needed an 80.9% reduction of her LDL-C levels to achieve the target. According to our national protocol regulating PCSK9i prescription, treatment with PCSK9i in primary prevention can be initiated only after six months of therapy with the maximum tolerated dose of statin plus ezetimibe. Hence, we started treatment with a high-intensity statin (rosuvastatin 40 mg) plus ezetimibe 10 mg once daily, but her LDL-C was still 198 mg/dl after 6 months of therapy. Therefore, on August 2021 the patient started treatment with a PCSK9i mAb (alirocumab 150 mg SC every 2 weeks). She had never received therapy with any other mAb before.

### Follow-up and outcomes

**Figure 1:**
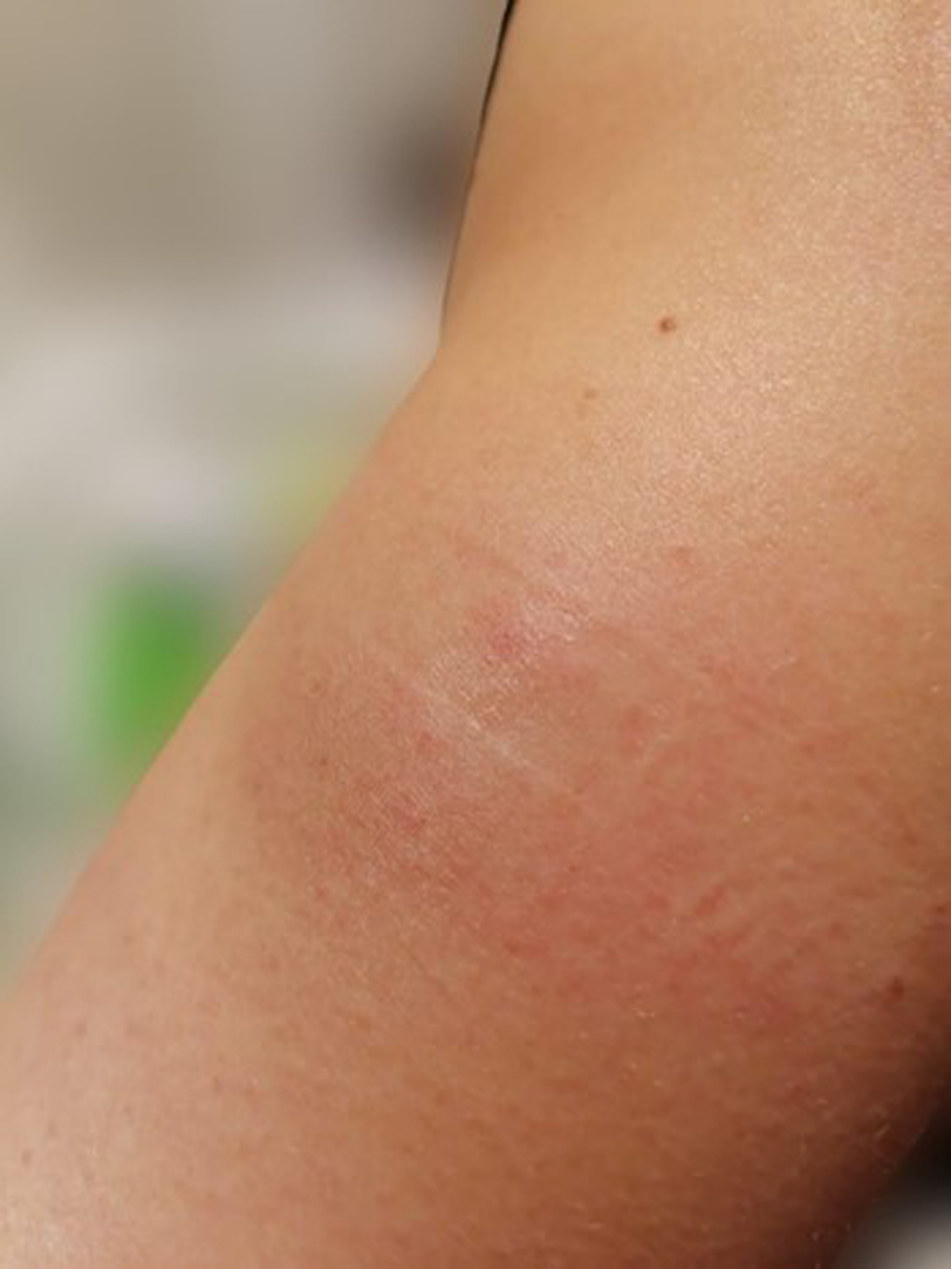
Erythematous and painful injection site reaction (ISR) developed 48 h after the second subcutaneous injection of alirocumab in the left arm.

**Figure 2:**
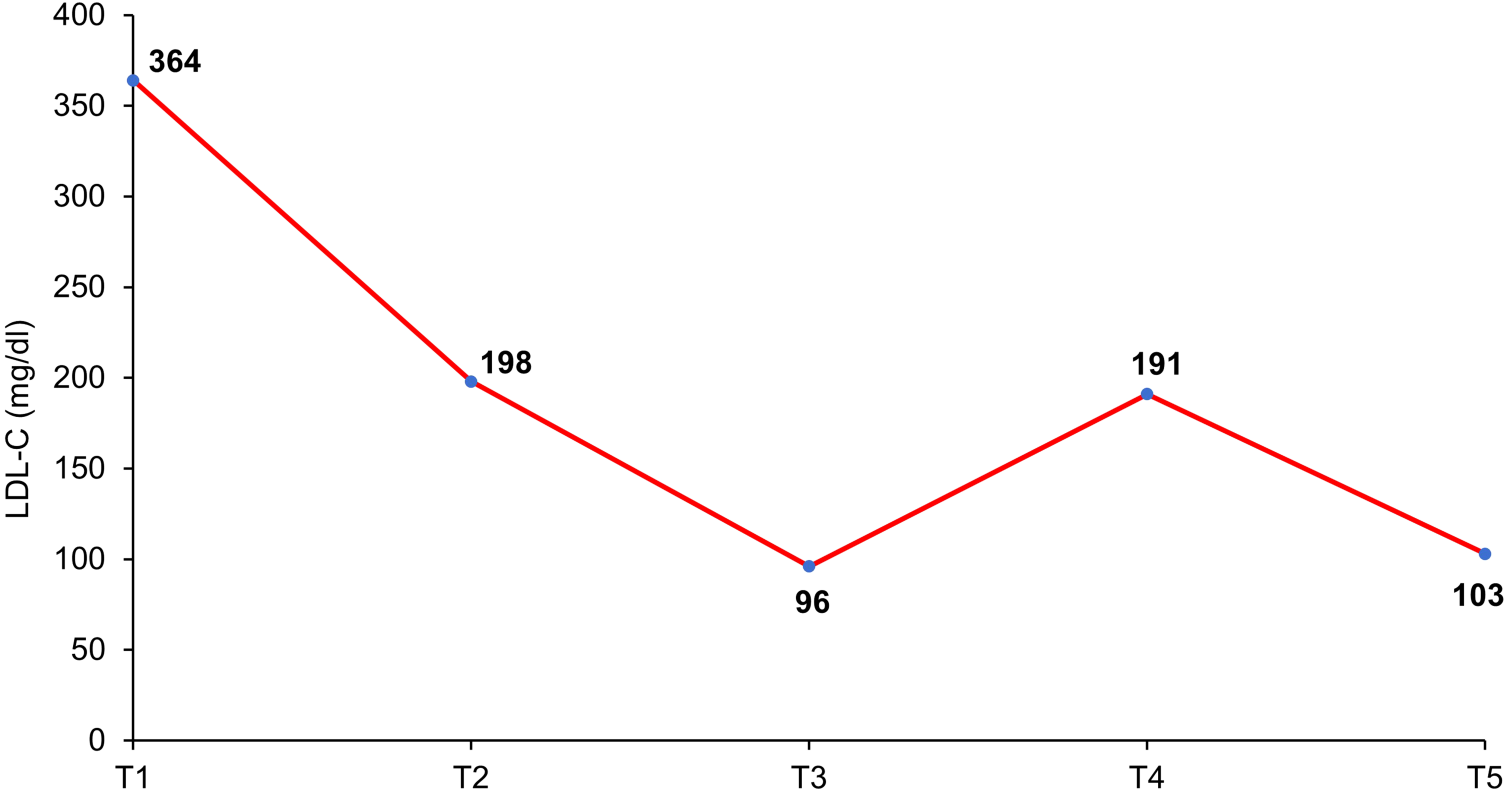
Timeline of LDL-C levels variations according to lipid-lowering therapy. T1: February 2021 (no lipid-lowering therapy); T2: August 2021 (6-month therapy with high-intensity statin plus ezetimibe); T3: September 2021 (1-month therapy with the addition of PCSK9-inhibitor mAb); T4: February 2022 (3-month PCSK9-inhibitor mAb withdrawal); T5: January 2023 (3-month therapy with the addition of inclisiran).

Forty-eight hours after the second SC injection of alirocumab in the left arm, the patient developed a painful and palpable ISR with an erythematous nodule of about 5 cm in major diameter (Figure 1), that resolved after 5 days of topical application of betamethasone. The skin of the injection site was intact, the administration was preceded by proper skin sterilization, the drug was at room temperature and no other injection had been performed in the area before the administration of PCSK9i; there was not a pro-inflammatory milieu, the patient did not suffer from allergic, dermatological, or immunological conditions and she had never experienced such a reaction after a drug injection previously. After two weeks, the administration was repeated in another injection site (contralateral arm), but an ISR with similar features was observed within 24 h from the injection. Based on the clinical features and the timing of onset, this ISR was consistent with a cell-mediated hypersensitivity reaction, or type IV reaction (14). Since the patient was at high risk for ASCVD, a mutual and informed decision was made to switch treatment from alirocumab 150 mg to evolocumab 140 mg. Unfortunately, despite pretreatment with oral prednisone, a similar ISR was observed within 24 h after the first SC injection of evolocumab in the abdomen. In each case, ISR resolved completely after 5 days. The patient denied both a biopsy and a skin test, but given the similarity of reactions to both drugs, we postulated the hypothesis of a cell-mediated hypersensitivity reaction elicited by an excipient contained in both mAbs [Praluent® [package insert]. Bridgewater, NJ:sanofi-aventis U.S. LLC; 2015; Repatha® [package insert]. Thousand Oaks, CA:Amgen Inc.; 2015] and known to be able to elicit such reactivity: polysorbate. Following this clinical suspicion, in agreement with the patient, who was no more able to tolerate the adverse event, treatment with PCSK9i mAbs was discontinued despite its demonstrated effectiveness. Indeed, PCSK9i mAbs therapy had lowered LDL-C to 96 mg/dl, while 3 months after withdrawal the LDL-C raised back to 191 mg/dl (Table 1 and Figure 2).

**Table 1:** Patient’s lipid values and clinical information during the study period.

**Time period**	**Lipid-lowering therapy**	**TC (mg/dl)**	**HDL-C (mg/dl)**	**Non-HDL-C (mg/dl)**	**LDL-C (mg/dl)**	**TG (mg/dl)**	**Lp(a) (mg/dl)**	**Clinical information**
February 2021	No therapy	445	54	391	364	124	178	HeFH-causing mutation in the LDLR gene; LDL-C polygenic score consistent with PHC; bilateral Achilles tendon xanthomas; family history of premature CHD
August 2021	6-month therapy with high-intensity statin plus ezetimibe	276	52	224	198	121	–	No adverse reactions to therapy
September 2021	1-month therapy with the addition of PCSK9i mAb	171	48	123	96	126	–	Painful and palpable ISR developing after the second injection of alirocumab and recurring after the third injection and after the switch to evolocumab
February 2022	3-month PCSK9i mAb withdrawal	278	55	223	191	155	–	Complete resolution of ISR
January 2023	3-month therapy with the addition of inclisiran	184	59	125	103	73	113	No adverse reactions to therapy

CHD, coronary heart disease; HeFH, heterozygous familial hypercholesterolemia; HDL-C, high-density lipoprotein-cholesterol; ISR, injection site reaction; LDL-C, low-density lipoprotein-cholesterol; LDLR, low-density lipoprotein receptor; Lp(a), lipoprotein(a); mAb, monoclonal antibody; PCSK9i, proprotein convertase subtilisin/kexin type 9-inhibitor; PHC, polygenic hypercholesterolemia; TC, total cholesterol; TG, triglycerides.

In October 2022, inclisiran has been approved in Italy for the treatment of severe hypercholesterolemia, offering a new complement to statin-based therapy. Since the siRNA inclisiran has a different formulation than that of mAbs (although administered by SC route as well) and does not contain polysorbate, we decided to start therapy with this new drug. The first SC administration of inclisiran was performed in October 2022 and a second one after 3 months, leading to no adverse reaction. Furthermore, three months after the first injection of inclisiran, the patient’s lipid panel was the following: TC 184 mg/dl, HDL-C 59 mg/dl, TG 73 mg/dl, LDL-C 103 mg/dl, Lp(a) 113 mg/dl (Table 1 and Figure 2).

## Discussion

Patients affected by FH have a significantly higher risk of premature CHD compared with the general population, but a timely diagnosis and early initiation of lipid-lowering therapy can even normalize life expectancy (15). Our patient had a pathogenic mutation in the LDLR gene and, additionally, she carried multiple LDL-C-raising SNPs. There is no consensus about the cut-off value of each polygenic score for the diagnosis of PHC. Unlike FH, PHC is not a dichotomous diagnosis but rather a continuum spectrum conferring CV risk in a dose-dependent manner (16). In the absence of any lipid-lowering therapy, our patient had higher LDL-C levels compared with her mother and a higher LDL-C score according to Talmud equation (11). We can hypothesize that several LDL-C-raising polymorphisms, in addition to the FH-causing monogenic mutation, could have contributed to such high LDL-C levels in our young patient. Therefore, our patient was burdened by a significant CV risk due to a monogenic hypercholesterolemia coupled with a further polygenic contribution. Indeed, patients with monogenic or polygenic hypercholesterolemia have a higher CV risk than those without a genetically determined cause, probably because they are exposed to a higher cumulative lifetime atherosclerotic burden (17).

Injection site reactions are the most common adverse events related to PCSK9i mAbs. They occurred in 6.1% of patients treated with alirocumab versus 4.1% in the control group receiving placebo, with a slightly lower percentage for evolocumab, without any significant difference between the two drugs regarding other safety and efficacy endpoints (18). Usually, these ISRs consist of erythema at the injection site, with possibility of warming, swelling and itching. The reaction may be immediate, although it usually appears within 24–48 h. Typically, ISRs due to mAbs are cell-mediated hypersensitivity reactions, i.e., type IV reaction, or type β immunogenicity (against therapeutic protein), according to a more recent classification of adverse reactions to biological drugs (19).

Our patient developed an erythematous and painful ISR of about 5 cm in major diameter (Figure 1) after 48 h following the second SC injection of alirocumab, resolving after topical application of betamethasone. Delayed timing of reaction confirmed a predominant role for T cells in the onset and maintenance of immune-mediated hypersensitivity (14). Given the similarity of reactions to both mAbs, we hypothesized a cell-mediated hypersensitivity reaction elicited by polysorbate, a common excipient of both alirocumab and evolocumab. Singh et al. described a patient who developed erythematous ISRs to both PCSK9i, with a skin test showing a reaction to polysorbate 80 (20). Polysorbates (PS20 and PS80) are almost universally used as excipients in biological therapies. Usually, excipients are used to stabilize a biological formulation, but this does not imply that they are inert substances (21). After the degradation of PS20 or PS80, protein aggregates can form, potentially enhancing product-related immunogenicity. Both PS80 and PS20 can directly activate complement (22) and their degradation products can function as haptens, eliciting a type IV immunological reactivity (23, 24). Furthermore, these degradation products can react with mAbs leading to the formation of reactive carbonyl adducts and antibodies against them (25). Fortunately, no cases of neutralizing antibodies against PCSK9i mAbs causing loss of efficacy in LDL-C lowering have ever been reported in literature so far. Another example of hypersensitivity due to polysorbate has been described after administration of erythropoietin. Patient developed generalized pruritus, erythema and orofacial angioedema, and an erythropoietin preparation without polysorbate had to be administered to continue therapy (26). Although ISRs to PCSK9i mAbs are usually transient, thus allowing treatment continuation under proper supervision (8), treatment with mAbs was discontinued in our case, because the patient was no longer able to tolerate the adverse event that recurred after every administration in an exacerbated way.

Inclisiran is a siRNA targeting hepatic PCSK9 synthesis, aiming at reducing circulating LDL-C, with the advantage of less frequent dosing regimen compared to PCSK9i (a single SC injection initially, then after 3 months and every 6 months thereafter). In the ORION-9, a double-blind trial which evaluated the efficacy and safety of inclisiran in patients with HeFH, LDL-C levels decreased by 39.7% from baseline to day 510 in the inclisiran group vs. 8.2% in the placebo group (*p* < 0.001), with a mean absolute reduction of 59 mg/dl. In addition, a further benefit of inclisiran therapy was due to a reduction of Lp(a) levels by 17.2% from baseline, contrasting thereby another independent risk factor for ASCVD (6). Surprisingly, our patient had a 36.5% reduction in Lp(a) levels (from 178 mg/dl to 113 mg/dl) after administration of inclisiran. In patients with ASCVD, the ORION-10 trial described a 51.3% reduction of LDL-C in the inclisiran group compared to a 1% change in the placebo group (*p* < 0.001). Even in a population with equivalent CV risk without a history of ASCVD (as the case of our patient), ORION-11 trial showed a greater percentage reduction of LDL-C in the inclisiran group compared to placebo (45.8% vs. 4%; *p* < 0.001) (27). ORION-9, −10 and −11 also evaluated the safety profile of inclisiran, describing ISRs as the primary adverse event, with a 5% incidence rate in the inclisiran group. Furthermore, in these trials antidrug antibodies have been found in a low percentage of patients, 2.0% of the samples from inclisiran-treated patients in the ORION-10 and 2.5% in ORION-11 (27), but no differences were found in the clinical efficacy and safety of inclisiran (28). The effect of LDL-C on the risk of ASCVD increases with increasing duration of exposure, as suggested by Mendelian randomization studies. Therefore, lipid-lowering treatment may reduce this risk the more the earlier it is started. Indeed, five years of treatment reduces the relative risk of ASCVD by ∼20%–25% per mmol/L of LDL-C reduction, while a 52-year exposure to lower LDL-C is expected to reduce ASCVD risk by ∼50%–55% per mmol/l of LDL-C reduction (29). However, ongoing studies will demonstrate whether the actual reduction in LDL-C levels found with inclisiran will translate into a reduction in CV events, as expected, and they will provide long-term information on the safety of this new drug.

Despite therapy with high-intensity statin, ezetimibe and inclisiran, leading to a 71.7% reduction in LDL-C levels, our patient did not reach the LDL-C goal according to her CV risk. It is already known that patients affected by FH may have a lower responsiveness to lipid-lowering therapies compared to patients not affected by genetically determined hypercholesterolemia (17).

The main limitation of this case report is the absence of a skin biopsy for histological examination and a skin test to obtain a definite diagnosis, due to the denied consent to perform these exams by the patient. Nevertheless, we believe that cell-mediated hypersensitivity to polysorbate is the most pathophysiological conceivable cause of the ISR.

### Patient perspective

This case report described a severe ISR after SC administration of both PCSK9i mAbs leading to treatment withdrawal, thus determining an increased risk of future ASCVD. Furthermore, this case report showed how the administration of inclisiran, an innovative treatment for hypercholesterolemia, proved to be a safe and effective therapeutic option in a patient at high CV risk who cannot achieve LDL-C goal despite maximal dose of high-intensity statin plus ezetimibe. Inclisiran is a new powerful weapon in patients affected by hypercholesterolemia regardless the presence of a genetically determined cause. In addition, inclisiran has to be administered by a healthcare worker, thus avoiding drug adherence-related issues. Last but not least, inclisiran was also able to reduce Lp(a) levels by over 36% in our patient, thus further improving the CV protection. Our patient was greatly relieved to have started an effective therapy in the absence of any adverse event. She is aware that her therapeutic goal has yet to be achieved; nevertheless, she has been informed that more lipid-lowering drugs, such as bempedoic acid, will soon become available in clinical practice, opening novel insights on dyslipidemia and CV risk management.

## Data Availability

The raw data supporting the conclusions of this article will be made available by the authors, without undue reservation.
